# Learning from the past and expecting the future in Parkinsonism: Dopaminergic influence on predictions about the timing of future events

**DOI:** 10.1016/j.neuropsychologia.2019.02.003

**Published:** 2019-04

**Authors:** Alessandro Tomassini, Thomas A. Pollak, Mark J. Edwards, Sven Bestmann

**Affiliations:** a MRC Cognition and Brain Sciences Unit, University of Cambridge, UK; b Department of Psychosis Studies, King's College London, UK; c Molecular and Clinical Sciences Research Institute, St George University of London, UK; d Sobell Department of Motor Neuroscience and Movement Disorders, University College London, UK

**Keywords:** Dopamine, Parkinson's Disease, Temporal processing, Temporal expectation, Motor preparation, Uncertainty, Drift-diffusion model

## Abstract

The prolonged reaction times seen in Parkinson's disease (PD) have been linked to a dopaminergic-dependent deficit in using prior information to prepare responses, but also have been explained by an altered temporal processing. However, an underlying cognitive mechanism linking dopamine, temporal processing and response preparation remains elusive.

To address this, we studied PD patients, with or without medication, and age-matched healthy individuals using a variable foreperiod task requiring speeded responses to a visual stimulus occurring at variable onset-times, with block-wise changes in the temporal predictability of visual stimuli.

Compared with controls, unmedicated patients showed impaired use of prior information to prepare their responses, as reflected by slower reaction times, regardless of the level of temporal predictability. Crucially, after dopamine administration normal performance was restored, with faster responses for high temporal predictability.

Using Bayesian hierarchical drift-diffusion modelling, we estimated the parameters that determine temporal preparation. In this theoretical framework, impaired temporal preparation under dopaminergic depletion was driven by inflexibly high decision boundaries (i.e. participants were always extremely cautious). This indexes high levels of uncertainty about temporal predictions irrespectively of stimulus onset predictability.

Our results suggest that dopaminergic depletion in PD affects the uncertainty of predictions about the timing of future events (temporal predictions), which are crucial for the anticipatory preparation of responses. Dopamine, which is affected in PD, controls the ability to predict the timing of future events.

## Highlights

• Parkinson’s patients cannot use prior temporal information to prepare motor responses.
• Temporal preparation is restored to normality by dopamine administration.
• Temporal preparation declines with increasing uncertainty about temporal predictions.
• Dopaminergic depletion linked to inflexibly high uncertainty in temporal predictions.
• Dopamine regulates the uncertainty of predictions about the timing of future events.

## 1 Introduction

Our ‘beliefs’ about the external world allow for generating predictions about the possible timing of future events (temporal predictions, Nobre et al., 2007). However, temporal predictions carry a degree of uncertainty (temporal uncertainty) that scales with the length and variability (predictability) of the delay preceding an event (foreperiod; Gibbon et al., 1997). When the onset of a stimulus is predictable, reaction times (RT) speed-up (Niemi and Näätänen, 1981). But when the length and/or variability of foreperiods increases, temporal predictions become more uncertain, leading to slower RTs (Klemmer, 1956).

The neurotransmitter dopamine (DA) plays a central role in temporal processing and the formation of temporal predictions (Coull et al., 2011, Meck, 1996). Along its role in encoding uncertainty about the occurrence of a stimulus, such as the delivery of a reward (de Lafuente and Romo, 2011), dopaminergic activity might also signal the uncertainty about when a stimulus occurs (e.g. time of reward delivery). For example, when long fixed foreperiods precede reward delivery (Bromberg-Martin et al., 2010, Nomoto et al., 2010), or when the foreperiods are variable (variable foreperiod), dopamine midbrain cells respond in relation to the temporal predictability of the reward delivery time (Pasquereau and Turner, 2015). Crucially, suppression of dopaminergic neurotransmission impairs temporal judgments (Soares et al., 2016) and the ability to form temporal predictions (Tomassini et al., 2015), possibly through a dopamine-dependent increase in uncertainty about the underlying temporal structure of events.

A role of dopamine for controlling temporal uncertainty (Fiorillo et al., 2003) suggests that dopamine depletion should impair the ability to form accurate temporal predictions (Friston et al., 2012). Here we tested this in Parkinson's disease (PD) patients as a model for DA depletion, to elucidate the role of DA in regulating the levels of temporal uncertainty. PD is a movement disorder characterized by degeneration of midbrain DA cells and among the cognitive impairments observed in PD patients are the inability to combine past experience to guide decisions (Perugini et al., 2016), along with altered processing of time durations.

**Fig. 1:**
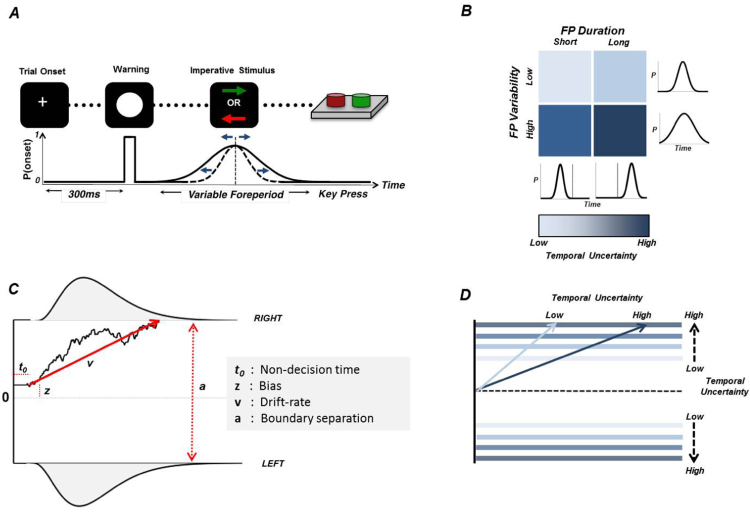
Variable foreperiod task and the drift-diffusion model. (a) Following a warning stimulus (white circle), the onset of the imperative stimulus (colored arrows) required participants to make a keypress as quickly as possible. The time between the warning and imperative stimulus (foreperiod) changed across trials following a Gaussian distribution. (b) Mean and standard deviation of the foreperiod distribution were constant throughout a block of trials. Four different distributions were used in a blocked factorial design: foreperiod duration (Short, Long) x foreperiod variability (Low, High). The formation of precise temporal predictions was thus mainly limited by temporal uncertainty, which increased with foreperiod duration and foreperiod variability. (c) Example of a trajectory of the drift-diffusion model. The two boundaries represent action-triggering boundaries for ‘LEFT’ and ‘RIGHT’ responses. The diffusion process, reflecting the accumulation of sensorial evidence, starts at a point, *z,* between the two boundaries and after a non-decision time, *t*_*0,*_ rising up to one of the two action-triggering boundaries with drift-rate, *v*. The predicted RT is the sum of the duration of the diffusion process and *t*_*0*_. (d) Hypothetical effects of temporal uncertainty on parameters of the model: temporal uncertainty may either modulate the sampling-rate of sensorial evidence accumulation (i.e. the drift-rate), increase the level of activation required to trigger an action (i.e. boundaries), or a combination of both. (For interpretation of the references to color in this figure legend, the reader is referred to the web version of this article.)

However, it is still unknown whether DA depletion in PD patients impairs the ability to correctly represent temporal uncertainty and hence to form temporal predictions about future events. This is crucial because some impairments of movement control that characterize the disorder have been attributed to either an inability to react to novel events, or an over-reliance on new sensory information (Galea et al., 2012, Perugini et al., 2016). We addressed this question by comparing medicated (PD-on) and unmedicated (PD-off) PD patients, and aged-matched controls using a variable foreperiod task that allows to probabilistically predicting the timing of the stimulus onset (Gibbon et al., 1997, Luce, 1986). We measured RT across a range of predictability levels by manipulating the mean and variance of the foreperiod distributions in a block-wise fashion, thus linking actual stimulus-onset predictability to experienced temporal uncertainty (Fig. 1A-B).

By fitting RTs to a drift-diffusion model (DDM), we sought to identify the specific decision processes relating to changes in temporal uncertainty and their sensitivity to DA depletion. We applied a Bayesian hierarchical estimation of DDM, which is particularly suited for studies involving patients where trial counts are necessarily low (Wiecki et al., 2013), and which has been successfully employed to investigate PD (Cavanagh et al., 2011, Herz et al., 2017, Zhang et al., 2016). We compared three principled variants of DDM that reflected competing hypotheses about the link between DA and temporal uncertainty. One variant posits that more certain temporal predictions about stimulus-onset are reflected by lower boundaries. This leads to faster response initiation and hence faster reaction times (Näätänen, 1970). Conversely, high temporal uncertainty is reflected by higher decision boundaries, leading to more cautious, and hence slower responses (Forstmann et al., 2010; Fig. 1D). An alternative model is that temporal uncertainty influences the ability to deploy attention (Nobre et al., 2007), as reflected by changes in the drift-rate of the accumulation (Forstmann et al., 2011). Higher drift-rates thus occur with high attentional engagement, and vice-versa. A third possibility is an interaction between drift-rate and boundary across conditions, suggesting that temporal uncertainty influences both response cautiousness and attention processes.

We tested this directly, hypothesizing that DDM parameters should vary across levels of temporal predictability reflecting changes in temporal uncertainty. We further hypothesized that dopaminergic depletion increases temporal uncertainty about temporal predictions, thus slowing response preparation (as indexed by slow RT), regardless of the actual predictability of stimulus onset. We further anticipate that the dopamine-induced deficit in estimating temporal uncertainty should be reflected by DDM parameters that do not adjust to changes of temporal predictability.

## 2 Materials and methods

### 2.1 Participants

Our 32 participants included sixteen patients with idiopathic PD and sixteen healthy controls. This sample size has proved powered enough in similar behavioural studies involving PD patients (Bellebaum et al., 2016, Hadj-Bouziane et al., 2012, Manohar and Husain, 2015).

PD patients and controls did not differ in age, education, gender distribution, and general measure of cognition (Table 1). Experimental protocols conformed to the guidelines of the Declaration of Helsinki and were approved by the research ethics committee of the Institute of Neurology at University College London.

**Table 1:** Demographic and clinical characteristics of participants.

	**Controls**	**Parkinson's patients**		**Statistics**	
			**t**	**df**	**p-value**
Gender (female / male)	10/6	9/7			
Age (years)	67.5 ± 7.3	66 ± 7.6	0.564	30	0.57
Education (years)	15.3 ± 3	14.6 ± 3.5	0.563	30	0.58
Time since diagnosis (years)	–	9.9 ± 4.9			
Time between testing sessions (days)	–	12.9 ± 5.2			
Time overnight withdrawal (OFF; hours)	–	14.7 ± 5.2			
LEU	–	712 ± 322			
*Hoehn-Yahr severity score*^a^	–				
OFF	–	2.0 ± 0.7			
ON	–	1.7 ± 0.7			
*MMSE*	29.2 ± 0.9				
OFF	–	28.5 ± 1.1	1.860	30	0.07
ON	–	28.6 ± 1.7	1.301	30	0.20
*HADS-A (Anxiety subscale)*	5.1 ± 3.7				
OFF	–	6.6 ± 3	− 1.274	30	0.21
ON	–	6.1 ± 3.1	− 0.881	30	0.38
*HADS-D (Depression subscale)*	3.3 ± 2.6				
OFF	–	5.0 ± 3.7	− 1.440	30	0.16
ON	–	4.6 ± 3.1	− 1.202	30	0.24
*UPDRS subscale III (motor)*^b^	–				
OFF	–	36.9 ± 13.3			
ON	–	22.5 ± 10.9			

Abbreviations: LEU, L-dopa equivalent units; MMSE, Mini-Mental State Examination; HADS, Hospital Anxiety and Depression Scale; UPDRS, Unified Parkinson's Disase Rating Scale. Table shows mean±SD.

a Hoehn-Yahr ON was significantly lower than Hoehn-Yahr OFF (t(15) = 2.61, p = 0.02).

b UPDRS ON was significantly lower than UPDRS OFF (t(15) = 6.19, p < 0.001).

Neurological and psychiatric symptoms were assessed using the Hoehn-Yahr Scale (Hoehn and Yahr, 1967), the Unified Parkinson's Disease Rating Scale (UPDRS; Lang and Fahn, 1989), the Hospital Anxiety and Depression Scale (HADS; Zigmond and Snaith, 1983). Both patients and healthy controls were administered the Mini Mental State Examination Scale (MMSE; Folstein et al., 1975); a pre-defined cut-off score of 25 represented a degree of cognitive impairment considered too great for participation. Excluding PD medications for the patient group, neither patients nor controls were under the effect of drugs potentially interfering with central dopamine levels during the testing period.

#### 2.1.1 Parkinson's disease patients

PD patients were tested on and off (877 ± 220 min withdrawn) their usual dopaminergic medication. All patients were treated with either L-dopa monotherapy (n = 3) or L-dopa in combination with dopamine agonist (n = 13). L-dopa equivalent units (mean 713 ± 322) were calculated as described elsewhere (Tomlinson et al., 2010). A comprehensive description of the patients’ demographic data is provided in Table 1. Patients were assessed twice (days between testing sessions 12.9 ± 5.2), once in medicated state (PD-on) and once after overnight withdrawal (mean 14.7 ± 5.2 h) of dopamine medication (PD-off). To control for learning effects, the order of the assessment was counterbalanced so that half (n = 8) of the patients omitted their medication for the first session and the other half for the second session. All the patients had a stable response to L-dopa and showed no sign of dyskinesia during the experiment.

#### 2.1.2 Healthy participants

Age-matched controls (six females, eight males; mean age 67.5 ± 7.3) had no history of neurological disorder and none of them were taking dopamine replacement medications.

### 2.2 Apparatus and procedures

#### 2.2.1 Apparatus

Stimuli were presented using MATLAB (The MathWorks, Natick, MA) and Cogent Graphics routines (http://www.vislab.ucl.ac.uk/cogent.php) on a 19-in. LCD display (refresh rate 60 Hz) controlled by a Dell Precision T3500 (Dell Computer Corp., Austin, TX).

#### 2.2.2 Behavioural procedure

We examined temporal preparation using a variable foreperiod task (Fig. 1a) in which the delay (foreperiod) between a warning stimulus (white circle) and an imperative stimulus (colored arrow) was varied across trials (Fig. 1A). Visual stimuli subtended approximately 5° of visual angle at a viewing distance of 60 cm. Participants had to respond to the appearance of the imperative stimulus as quickly and as accurately as possible by pressing either the left or the right arrow key with their dominant hand (Right handed patients: 15/16; Right handed controls: 14/16). Here, the reaction times (RT) indicate the degree of preparation, such that greater preparation generates faster reaction times (Niemi and Näätänen, 1981).

On each trial, the foreperiod duration was randomly sampled from a truncated Gaussian distribution (Fig. 1B). In a blocked 2 × 2 factorial design, we manipulated mean foreperiod duration (Short: 1500 ms; Long: 3000 ms) and foreperiod variability (i.e. standard deviation; Low: 100 ms; High: 600 ms). The two distributions for short foreperiods were truncated at 500 ms, here considered as the minimum time required for preparation (Hackley et al., 2009), and at 2500 ms (longest foreperiod) to preserve the distribution's symmetry. To ensure comparable standard deviations between short and long distributions, both tails were also truncated to the long distribution at 2000 ms and 4000 ms.

Each experimental session consisted of 4 blocks of 120 trials each, separated by a short rest. A training block of 40 trials was conducted prior to the main experiment. The training block was identical to the experimental counterparts, but with the foreperiod sampled from an exponential (non-ageing) distribution with mean 1000 ms (Niemi and Näätänen, 1981). The order of conditions (blocks) was balanced across sessions and participants following a Latin square design. The whole experiment comprised one session for the controls and two sessions, one on and another off medication, for the PD patients.

### 2.3 Data analysis

#### 2.3.1 Reaction times

RT were calculated as the delay between the onset of the imperative stimulus and the key press. Responses shorter than 100 ms or exceeding the individual median RT by more than 3 median absolute deviations (MAD) were considered invalid and excluded from further analysis. A Poisson regression characterized the relationship between RT and temporal predictability in terms of intercept and slope of a linear function fitted to the RT of each participant (Banca et al., 2014):

(1) $$\log\left(E\left(\mathrm{RT}|\mathrm{Tp}\right)\right)=\beta_{0}+\beta_{1}\mathrm{Tp}$$

*Tp* (temporal predictability) varies from 4 (high) to 1 (low). The intercept (*β*_*0*_) corresponds to the expected RT when temporal predictability is highest and quantifies absolute differences in temporal uncertainty (indexed by RT) between groups not attributable to our manipulation. The slope (*β*_*1*_) measures the sensitivity of RT to variations in temporal predictability and represents the strength of the relationship between subjective temporal uncertainty and temporal predictability. Specifically, positive slopes indicate that temporal uncertainty increases when reducing temporal predictability whereas slopes close to zero indicate no relationship.

In addition, we tested whether PD-off patients were able to modulate their RTs with the passage of time (foreperiod-effect; Luce, 1986) showing faster RT for foreperiods near the end of the trial than for early ones. A linear regression between foreperiod's length and RT quantified the foreperiod-effect between groups, with negative slopes indicating faster RT for longer foreperiods. This was to confirm that performance in PD-off was not determined by impaired motor preparation or/and temporal processing.

#### 2.3.2 Drift-diffusion modelling

In the drift-diffusion models(DDM) the decision process is described by four parameters (Fig. 1C): the separation between the two decisions boundaries (*b*) corresponding to the two choice alternatives (e.g. left vs right button), the rate of activity accumulation arising towards the boundaries (v; drift rate), a *priori* bias towards one of the two decisions (z), and non-decision time t_0_ representing the time used for stimulus encoding and response execution latencies. The model predicts the RT for each alternative as the latency for the accumulating activity to reach the corresponding boundary.

Here, we used a *stimulus-coding* approach where the lower and upper boundary correspond to left and right responses, respectively.

We applied a hierarchical Bayesian approach (http://ski.clps.brown.edu/hddm_docs/, Wiecki et al., 2013) to fit the model to the empirical RT. The hierarchical approach treats participants as random variables drawn from the group-level distribution, and uses Bayesian statistics to estimate the posterior distribution of the DDM parameters at the group-level, while accounting for differences at the participant-level (Wiecki et al., 2013). This approach is robust in estimating parameters with limited data, and thus particularly well suited for studies involving clinical populations given the substantial constraints on the duration of the task for patients (Zhang et al., 2016).

To test such hypotheses, we first compared 3 variants of the DDM varying systematically whether only the boundaries (Model 1), the drift-rate (Model 2) or a combination of both (Model 3) were allowed to change between levels of temporal predictability. We did not expect *a priori* biases since the two alternatives were presented in random order, and counterbalanced by condition. Non-decision time was kept constant across levels of temporal predictability, given prior evidence of a dissociation between motor performance (i.e. kinematic parameters) and temporal uncertainty (Tomassini et al., 2015; Pasquereau and Turner, 2015), but was allowed to vary across groups to reflect dopamine-related changes. All parameters were estimated separately for each group.

For all variants, Markov Chain Monte Carlo simulations were run to generate 50,000 samples from the estimated joint posterior parameter distribution and the first 5000 samples were discarded as burn-in. Convergence was assessed by visual inspection of the Markov chains and by calculating the R-hat Gelman-Rubin statistics (Krypotos et al., 2015).

**Fig. A1:**
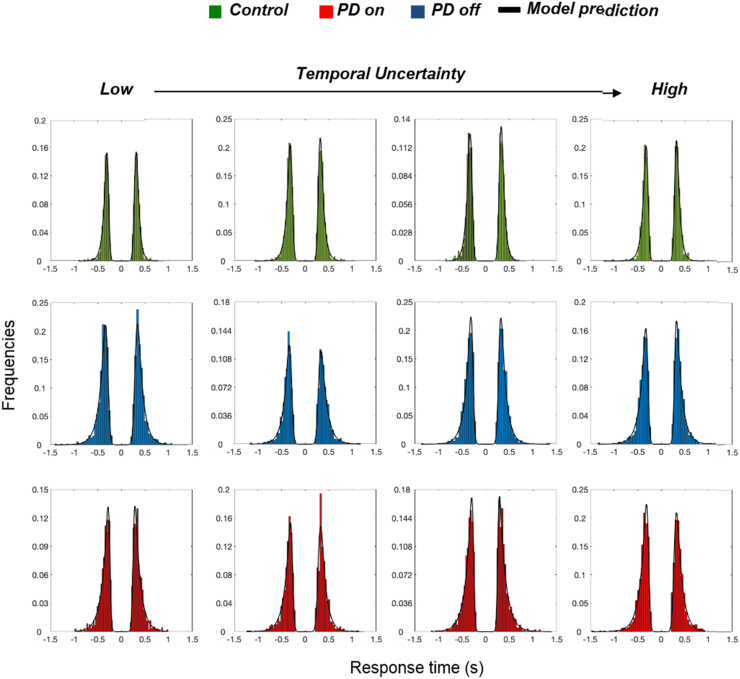
Posterior predictive data distribution from the winning model. The distributions along the positive and negative x-axis show the latency distribution for the left and right response, respectively. Each panel shows the normalized histograms of the observed data and the model predictions (black lines). The model predictions were generated by averaging 500 simulations of the same amount of model predicted data as observed in the experiment using posterior parameter estimates. Each row shows the distribution for each group (color-coded) whereas the columns are arranged according to the level of temporal uncertainty. The plots show a good agreement between the observed data and the model predictions across temporal uncertainty levels in all three groups.

Model comparison was performed by comparing the deviance information criterion (DIC) value of each variant (lowest DIC indicates the best fit; Spiegelhalter, 2002), and revealed that our data were best fitted when only the boundary parameter varied across conditions (i.e. Model 1; See results below for more details; See Table A1 in the appendix for the parameters of the winning model). Hence, from here onward, we will only refer to this model. To further evaluate the quality of model fitting, we ran posterior predictive checks by averaging 500 simulations generated from the model's posterior to confirm it could reliably reproduce pattern in the observed data (see appendix Table A2 and appendix Fig. A1 for results of posterior checks).

We predicted that impaired behavioural performance should arise from the inability to correctly map temporal uncertainty to different levels of temporal predictability. Following the same logic of the RT analysis, we characterized the relationship between temporal uncertainty (indexed by the boundary parameter) and temporal predictability in terms of intercept (*β*_*0*_) and slope (*β*_*1*_) of a linear function fitted to the estimated boundary of each participant. Since the intercept occurs when the predictability of stimulus-onset is highest, it represents the expected boundary's value when temporal predictability is highest and provides insight on the ‘baseline’ levels of temporal uncertainty across groups. The slope quantifies the modulatory effect of temporal predictability on temporal uncertainty, as reflected by the boundary parameter, with slopes close to zero indicating a weak modulatory effect. The anonymised behavioural data, and source code used for the analyses can be found at https://github.com/ale-tom/PD_HDDM.

#### 2.3.3 Statistical analyses

Within group effects of medication (PD-on, PD-off) were assessed using one-tailed paired *t-*tests. Between groups effects were tested using one-way ANOVAs with post-hoc planned comparisons (Controls vs PD-on; Controls vs PD-off; Keppel, 1991). We report η^2^ as a measure of main effect size and Cohen's d_z_ (Cohen, 1988) for the effect size of *t*-test comparisons. For all analyses, level of statistical significance was fixed at 0.05.

Statistical inference on model parameters was made by comparing the proportion (q) of the posterior distribution of each parameter that overlaps between groups. Significance was assigned if less of the 16.7% (q<0.0167) of the distributions overlapped, corresponding to q= 0.05 (Wiecki et al., 2013) after applying Bonferroni correction (Banca et al., 2014).

## 3 Results

### 3.1 Clinical assessments

Patients were in the mild to moderate stage of the disease, with more pronounced symptoms when in the off state (Hoehn and Yahr scale, t_15_ = 2.61, *p* = 0.02). As expected, scores of the motor section of the UPDRS were significantly higher (t_15_ = 6.19, *p* < 0.001) when measured off compared to on medication. None of the patients had dementia diagnoses (MMSE scores > 25) and none suffered from clinical depression (HADS scores <7). See Table 1 for further details.

### 3.2 Basic reaction time effects

Participants performed the task without difficulty as shown by the small overall proportion of excluded trials (control: 2.5% PD-on: 8.6%, PD-off: 9.3% of responses). Less errors were made by the control group (mean 3% ± 2%) compared with the PD-off (mean 18% ± 25%)and PD-on groups (mean 19% ± 24%), although patients and control subjects did not differ significantly (ANOVA, F(2,44) = 3.14, p = 0.053). The data from one healthy volunteer were excluded due to technical problems during data acquisition.

**Fig. 2:**
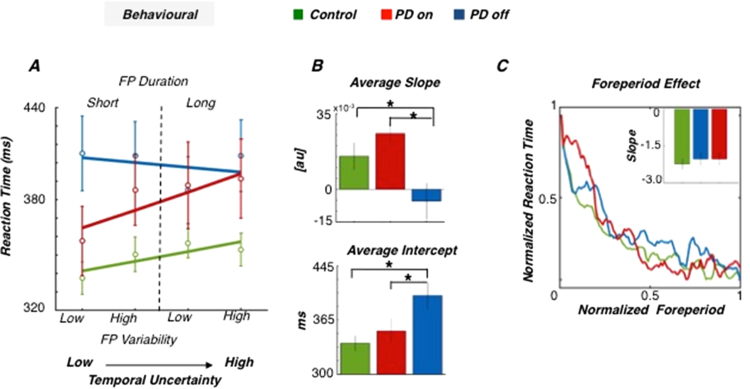
Behavioural results (a) Modulation of RTs by temporal predictability. Average RTs are plotted against foreperiod variability (bottom abscissa) and segregated by foreperiod duration (upper abscissa) in controls (green), PD-on (red) and PD-off (blue) patients. (b) Poisson regression. The relationship between RTs and temporal predictability were characterized in terms of intercept (β_0_) and slope (β_1_) of a linear function fitted to the RTs of each participant. * p < 0.05; Error bars represent ± 1 SEM. (c) Foreperiod analysis. RTs were averaged across subjects and conditions and plotted as a function of foreperiod duration. RTs were normalized and smoothed only for illustrative purposes. The inset barplot shows the slopes of the linear regression between RTs and foreperiod duration. Negative slopes indicate a normal foreperiod effect. No significant difference between groups was detected.

Our behavioural results show that although participants were not aware of the different foreperiod distributions, PD-on and controls were able to learn the underlying temporal structure of the task and used temporal predictions in anticipation of forthcoming stimuli (Fig. 2 A–B). Indeed, our Poisson regression revealed that, in controls, RTs were modulated by the temporal predictability of stimulus-onset (slopes of Poisson regression, one-sample *t*-test against zero: control: t_15_ =2.588, p = 0.002) with slower RT when the temporal predictability was lowest. By contrast, PD-off patients showed slow RT regardless of the level of predictability (slope of Poisson regression, one-sample *t*-test against zero; PD-off: t_15_ = 0.562, p = 0.582; between-group comparison: *β*_*1*_: F_2,46_ =5.557, p = 0.007, η^2^ = 0.187; controls vs PD-off post-hoc; t_24.33_ =0.52, p = 0.002, d_z_ =1.3; corrected for heteroscedasticity). Crucially, after dopaminergic administration, patients’ performance was restored to normality (regression slope. One-sample *t*-test against zero: PD-on: t_15_ =5.369, p < 0.001; PD-on vs PD-off: paired *t*-test: t_15_ =3.767, p = 0.001, d_z_ =0.96; Controls vs PD-on: two-sample *t*-test: t_29_ =1.310, p = 0.2). Since we assume that the changes in RT across levels of predictability index changes in temporal uncertainty, our data suggests that temporal uncertainty has saturated after DA depletion.

Poisson regression also revealed significant differences in the RT intercept (*β*_*0*_: F_2,46_ =4.696, p = 0.014, η^2^ = 0.173) between groups. The intercept in Fig. 2A-B quantifies RTs when the stimulus onset can be easily predicted and is consistent with PD-off patients being overall slower than PD-on (paired *t*-test; t_15_ =3.767, p = 0.001, d_z_ =0.96) and controls (post-hoc; t_24.33_ =0.52, p = 0.002, d_z_ =1.3; corrected for heteroscedasticity). However, foreperiod analyses confirmed that PD-off were still able to modulate their response speed depending on the passage of time. Foreperiod effects were marginally weaker for PD patients (both on and off medication) than controls (see inset Fig. 2C), however such differences were not statistically significant (two sample *t*-test: control vs PD-on, t_29_ = −0.572 p = 0.57; Control vs PD-off, t_29_ = −0.277, p = 0.78; paired *t*-test: PD-on vs PD-off: t_15_ = 1.398, p = 0.19. All comparisons Bonferroni corrected). Thus, the lack of difference in RT across predictability levels contradistinctive of DA depletion cannot be attributed to a general impairment of motor preparation or to experience the passage of time.

Together these results indicate that PD patients were not per se unable to form temporal predictions, but that this ability requires sufficiently restored levels of DA, as after administration of dopamine medication (see Table 2 for an overview).

**Table 2:** Behavioural and modelling results.

					**Statistics**		
	**Parameters**	**Control**	**PD-on**	**PD-off**	**Control vs PD-on**	**Control vs PD-off**	**PD-on vs PD-off**
**Behavioural**	Intercept (β_0_)	334 ± 1.11	354 ± 1.18	395 ± 1.20	p = 0.15	p = 0.002*	p = 0.001*
	Slope (β_1_)	0.015 ± 0.02	0.023 ± 0.02	− 0.004 ± 0.03	p = 0.18	p = 0.014*	p = 0.002*
**Model**	Intercept (β_0_)	0.869 ± 0.16	0.931 ± 0.10	1.065 ± 0.14	p = 0.21	p < 0.001*	p = 0.006*
	Slope (β_1_)	0.040 ± 0.05	0.059 ± 0.05	0.001 ± 0.05	p = 0.15	p = 0.015*	p = 0.003*

Table shows mean±SD.

* p < 0.05.

### 3.3 Drift-diffusion model

**Fig. 3:**
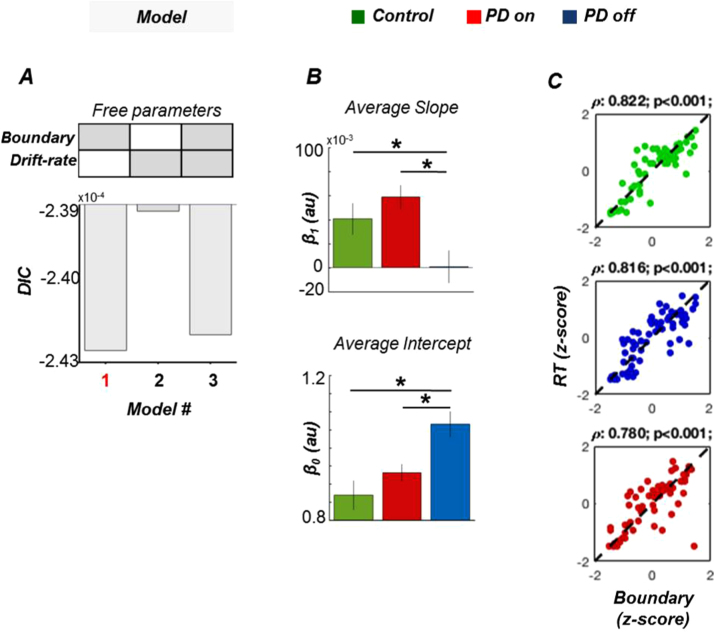
Model comparison and hierarchical drift-diffusion modelling of variable foreperiod task (a) Model comparison. The deviance information criterion (DIC) between the best fitting model (Model 1 - free parameter: boundary) and the two alternative models (Model 2 - drift-rate; Model 3 – boundary and drift rate) is shown. (b) Influence of predictability on evidence accumulation. Intercept and slope of a linear function were fitted to the estimated boundary of each participant. (c) Model fit. Estimated boundary values were strongly correlated to empirical RT averaged across trials within each predictability level (Spearman correlation); * p < 0.05; Error bars represent ± 1 SEM.

Changes in boundary separation alone were able to account for changes in RTs across different levels of temporal predictability and our experimental groups, as reflected by lowest DIC values for this model (Model 1; Fig. 3A). This result shows that the estimated boundary can be considered as a proxy measure of the temporal uncertainty experienced by the subject. Furthermore, no significant differences between groups were observed in either drift-rate (Control vs PD-on: q = 0.86; Control vs PD-off: q = 0.29; PD-on vs PD-off: q = 0.03) and non-decision time (Control vs PD-on: q = 0.31; Control vs PD-off: q = 0.73; PD-on vs PD-off: q = 0.89). These results confirm that our behavioural observations on PD-off did not result from an unspecific effect on alertness or the ability to move.

Following the same logic of the behavioural analysis, we characterized the relationship between temporal predictability and DDM boundaries in terms of intercept (*β*_*0*_) and slope (*β*_*1*_) of a linear function fitted to the estimated boundaries of each participant. The results from the linear fitting of boundaries paralleled the results from the behavioural analysis (Fig. 3). Both intercept (F_2,46_ =8.497, p = 0.001, η^2^ = 0.278) and slope (F_2,46_ =5.856, p = 0.006, η^2^ = 0.209) differed significantly between groups. Intercepts (*β*_*0*_) were significantly larger in PD-off, compared to PD-on (paired *t*-test; t_15_ =2.826, p = 0.006, d_z_ =0.71) and controls (post-hoc: t_44_ =4.013, p < 0.001, d_z_ =1), suggesting high levels of temporal uncertainty after dopamine depletion even when stimulus-onset was highly predictable. Slopes (*β*_*1*_) were significantly smaller in PD-off relative to PD-on (paired *t*-test; t_15_ =3.202, p = 0.003, d_z_ =1.23) and controls (post-hoc: t_44_ = 2.259, p = 0.015, d_z_ =0.81). There were no significant differences between PD-on and controls (*β*_*0*_: t_1.261_, p = 0.21 *β*_*1*_: t_1.035_, p = 0.15). The lack of difference between controls and PD-on groups suggests that a widening of decision boundaries along with a failure to adjust to changes in temporal predictability result from DA depletion.

A strong correspondence between estimated boundary values and the empirical RT (Fig. 3C; Control: rho = 0.82, p < 0.001; PD-on: rho = 0.78, p < 0.001; PD-off: rho = 0.81, p < 0.001) confirmed that the observed behavioural impairments were well explained by changes in the sole boundary parameter of the DDM (see Table 2 for a comparison).

Finally, the goodness of fit of the regression analyses was first quantified for each individual using Pearson's correlation coefficient and then Fisher transformed to meet distributional assumptions for parametric tests across groups. A one-way ANOVA on the Fisher-transformed correlation coefficients showed no difference in the goodness of fit across groups for neither empirical reaction times (F(2, 44) < 0.001, p = 1.00; mean R^2^ ± stdev: Controls = 0.83 ± 0.004, PD-Off = 0.83 ± 0.004, PD-On = 0.83 ± 0.009) nor model boundary parameter (F(2, 44) = 1.07, p = 0.35; mean R^2^ ± stdev: Controls = 0.84 ± 0.007, PD-Off = 0.83 ± 0.007, PD-On = 0.84 ± 0.005).

## 4 Discussion

Parkinson's disease (PD) is characterized by an impaired use of prior information to guide perceptual (Perugini et al., 2016) and value-based decisions (Frank et al., 2004; Shiner et al., 2012). Here we demonstrate that after DA medication withdrawal, PD patients are impaired when using prior experience about the time of occurrence of events to prepare responses in anticipation of an event. Critically, dopamine administration restores performance to levels comparable to the control group.

PD is a movement disorder often accompanied by bradykinesia (Sheridan and Flowers, 1990). A general inability to produce fast movements in PD-off patients could alternatively explain our results. However, most previous studies (Girotti et al., 1986, Jahanshahi et al., 1992, Starkstein et al., 1989; but cfr Zappia et al., 1994) have shown no difference in simple motor RT prior and after medication. Furthermore, PD-Off patients were able to modulate their speed showing increasing motor preparation with the passage of time. This suggests that bradykinetic symptoms in PD may result from a change in implicit ‘motor motivation’, as opposed to a general inability to move fast (Mazzoni et al., 2007). In previous work (Tomassini et al., 2015) adopting the same temporal manipulation, dopamine blockage in healthy participants produced strikingly similar results on temporal processing as observed here, without affecting the speed of reaching movements. In the present study, we furthermore observed that non-decision times estimated by the DDM (accounting for perceptual and motor processes) did not differ between groups. Differences in performance across groups were therefore neither driven by dopamine-related inability to produce fast movements, nor by deficits in temporal processing per se. Instead, our results indicate that DA depletion impairs the integration of prior temporal information for the preparation of actions.

Here we used a Bayesian framework as a theoretically grounded way to solve problems in the presence of uncertainty (Körding and Wolpert, 2006), and in which to explain the impairments observed in non-medicated patients. Within this framework, decisions rely more on more precise (i.e. less uncertain) sources of information (Friston et al., 2012, Mamassian and Landy, 2010, Stocker and Simoncelli, 2006). More specifically, in conditions of high temporal predictability participants will form reliable prior-beliefs about the most likely time of a stimulus (Acerbi et al., 2012, Ahrens and Sahani, 2011, Jazayeri and Shadlen, 2010), and use that information to prepare their responses. When reducing this predictability, priors become more uncertain and thus an optimal behaviour would require up-weighting the actual appearance of the stimulus rather than relying on one's temporal predictions. Accordingly, if dopamine depletion causes the overestimation of uncertainty about temporal predictions, a Bayesian agent will inflexibly rely on the external stimulus to determine when to make a response (Friston, 2014). This interpretation agrees with work (Perugini et al., 2016) suggesting that PD patients rely less on prior information to make decisions. Conversely, dopaminergic up-regulation should result in a bias towards prior expectations (Cassidy et al., 2018). Hallucinations, a cardinal feature of schizophrenia, are known to depend on excessive striatal dopamine (Weinstein et al., 2017). A Bayesian model of hallucinations posits that dopaminergic up-regulation can lead to a systematic underestimation of the uncertainty of predictions resulting in hallucinatory percepts reflecting excessive biases toward sensory expectations (Friston, 2005). Such idea could also be extended to temporal predictions, so that a Bayesian agent under hyper-dopaminergic state would inflexibly rely on *a priori* expectations to determine when to make a response. Future work on schizophrenic patients or pharmacological challenge (e.g. amphetamine) could assess such hypothesis.

The boundary parameter of our model captured the internal uncertainty about temporal predictions: Large boundaries under conditions of high temporal uncertainty index more cautious, and thus slower, responses (Forstmann et al., 2010). In agreement with our results, the effect of dopaminergic depletion on changes in DDM boundaries has been linked to basal ganglia function (Bogacz et al., 2010, Forstmann et al., 2008, Frank and O’Reilly, 2006, Hanks et al., 2014, Heitz and Schall, 2012, Herz et al., 2017). Accordingly, inflexibly large boundaries in PD-off indicate a dopamine-sensitive increase in uncertainty about when a stimulus is bound to appear (Tomassini et al., 2015). These results agree with previous work demonstrating a failure in adjusting the amount of sensory evidence needed to make a decision in PD (Perugini et al., 2016). They further echo earlier findings using healthy ageing as model for dopamine depletion, demonstrating inflexible decision boundaries in older relative to younger adults (Forstmann et al., 2011) performing speed-accuracy-trade-off tasks.

In older adults, adopting conservative decision criteria is presumed to be a compensatory strategy to prevent errors (Ratcliff et al., 2007, and references therein). One possible interpretation to our results is that dopamine-deficient individuals try to balance their performance by reducing temporal uncertainty with associated costs in terms of RT. In other words, an agent accumulates as much information as possible to reduce its internal uncertainty (Tajima et al., 2016), at the cost of slower responses.

In conclusion, we show that DA depletion increases the subjective levels of temporal uncertainty damaging the ability to use prior information in order to prepare to future events.

A better understanding of the mechanism linking temporal uncertainty and motor preparation may enable new therapeutic approaches to deal with impairments of movement control that characterize PD.

## Figures and Tables

**Table A1:** Model parameters.

**Parameters**	**Control**	**PD-on**	**PD-off**
Boundary (a)	0.9709 ± 0.15	1.078 ± 0.14	1.067 ± 0.12
a standard deviation	0.1455 ± 0.01	0.1455 ± 0.01	0.1455 ± 0.01
Drift-rate (v)	0.026 ± 0.039	0.085 ± 0.033	0.002 ± 0.032
v standard deviation	0.0331 ± 0.023	0.0331 ± 0.023	0.0331 ± 0.023
Non decision time (t0)	0.232 ± 0.009	0.226 ± 0.008	0.240 ± 0.009
t0 standard deviation	0.0353 ± 0.004	0.0353 ± 0.004	0.0353 ± 0.004
Bias (z)	0.70 ± 0.008	0.70 ± 0.008	0.70 ± 0.008
z standard deviation	0.143 ± 0.032	0.143 ± 0.032	0.143 ± 0.032

Table shows mean±SD.

**Table A2:** Posterior predictive checks.

		**Reaction times (sec)**			
		**Observed**	**Predicted**	**MSE**	**Credible**
**Left stimulus**	Mean	0.372	0.494	0.031	Yes
	Std	0.105	0.202	0.016	Yes
	10th quantile	0.271	0.316	0.007	Yes
	30th quantile	0.315	0.376	0.012	Yes
	50th quantile	0.35	0.431	0.021	Yes
	70th quantile	0.395	0.521	0.042	Yes
	90th quantile	0.494	0.742	0.111	Yes
**Right stimulus**	Mean	0.376	0.494	0.03	Yes
	Std	0.111	0.202	0.008	Yes
	10th quantile	0.272	0.316	0.001	Yes
	30th quantile	0.316	0.367	0.002	Yes
	50th quantile	0.352	0.431	0.006	Yes
	70th quantile	0.397	0.531	0.018	Yes
	90th quantile	0.502	0.744	0.058	Yes

## Appendix

See Table A1

Model parameters for the winning model

See Table A2

Comparison between statistics of the observed RT and statistics of the data predicted by the winning model. The mean-squared error (MSE) is a measure of how far the summary statistics of the observed data differ from the predicted data. MSE values close to zero indicate a good match between observed and simulated data. The Credible column shows whether the predicted data lie within the 95% credible interval from the observed data.

See Fig. A1
